# *Aloe vera*-Based Polymeric Network: A Promising Approach for Sustained Drug Delivery, Development, Characterization, and In Vitro Evaluation

**DOI:** 10.3390/gels9060474

**Published:** 2023-06-08

**Authors:** Arshad Mahmood, Alia Erum, Ume Ruqia Tulain, Sharmeen Shafiq, Nadia Shamshad Malik, Sidra, Muhammad Tariq Khan, Mohammed S. Alqahtani

**Affiliations:** 1 Faculty of Pharmacy, Al Ain University, Abu Dhabi Campus, Abu Dhabi P.O. Box 112612, United Arab Emirates; 2 AAU Health and Biomedical Research Center (HBRC), Al Ain University, Abu Dhabi P.O. Box 112612, United Arab Emirates; 3 Faculty of Pharmacy, University of Sargodha, Sargodha 40100, Pakistan; 4 Faculty of Pharmacy, Capital University of Science and Technology, Islamabad 45800, Pakistan; 5 Nanobiotechnology Unit, Department of Pharmaceutics, College of Pharmacy, King Saud University, Riyadh 11362, Saudi Arabia

**Keywords:** *Aloe vera*, polymeric network, acrylamide, thiocolchicoside

## Abstract

The present study was conducted to fabricate and characterize mucilage-based polymeric networks of *Aloe vera* for controlled drug release. *Aloe vera* mucilage was used to develop a polymeric network via the free-radical polymerization method using potassium persulphate as the initiator, N′ N′-Methylene bisacrylamide as the crosslinker, and acrylamide as the monomer. Using varying concentrations of *Aloe vera* mucilage, crosslinker, and monomer, we developed different formulations. Swelling studies were conducted at pH 1.2 and 7.4. Concentrations of polymer, monomer, and crosslinker were optimized as a function of swelling. Porosity and gel content were calculated for all samples. FTIR, SEM, XRD, TGA, and DSC studies were conducted for the characterization of polymeric networks. Thiocolchicoside was used as a model drug to study the in vitro release in acidic and alkaline pH. Various kinetics models were applied by using a DD solver. Increasing content of monomer and crosslinker swelling, porosity, and drug release decreased while gel content increased. An increase in *Aloe vera* mucilage concentration promotes swelling, porosity, and drug release of the polymeric network but decreases gel content. The FTIR study confirmed the formation of crosslinked networks. SEM indicated that the polymeric network had a porous structure. DSC and XRD studies indicated the entrapment of drugs inside the polymeric networks in amorphous form. The analytical method was validated according to ICH guidelines in terms of linearity, range, LOD, LOQ, accuracy, precision, and robustness. Analysis of drug release mechanism revealed Fickian behavior of all formulations. All these results indicated that the M1 formulation was considered to be the best polymeric network formulation in terms of sustaining drug release patterns.

## 1. Introduction

Over the years, a significant amount of research has focused on the development of novel drug delivery systems. Examples of such systems include liposomes, nanoparticles, microneedles, hydrogels, implantable drug delivery systems, and micro/nanostructured carriers. Currently, there is a growing trend among researchers to explore dosage forms derived from natural polymers, driven by their reduced toxicity and enhanced biocompatibility. The role of a drug delivery system is pivotal in regulating the release profile of a drug over an extended period by extending its residence time within the gastrointestinal tract (GIT). This approach enables controlled and sustained drug release, leading to improved patient adherence and heightened therapeutic efficacy. By reducing the frequency of daily single doses, drug therapy can be optimized to better align with patients’ requirements and treatment regimens [1].

Polymers are composed of long chains of macromolecules, which can be modified by incorporating various functional groups. This allows for tailoring their properties for specific applications by combining them with other substances of both high and low molecular weights. Synthetic as well as natural polymers find application in the pharmaceutical and food industries [2]. However, there has been a notable surge in interest regarding the utilization of natural polymers in various pharmaceutical applications. These polymers find extensive use as disintegrants, binders, and diluents in tablet formulations, thickeners in oral liquids, protective colloids in suspensions, gelling agents in gels, and bases in suppositories, as well as in matrix-controlled systems, film coating agents, buccal films, microspheres, and nanoparticles. Both natural and synthetic polymers are available for these applications; however, natural polymers possess distinct advantages over their synthetic counterparts. They are cost-effective, easily accessible, inert, nontoxic, biocompatible, and biodegradable [3]. Overall, the utilization of natural polymers in pharmaceutical formulations offers significant advantages over synthetic alternatives, making them a preferred choice in various drug delivery systems and formulations [4].

*Aloe vera*, scientifically known as *Aloe barbadensis*, belongs to the Liliaceae family. Among the more than 300 species of Aloe plants, *A. barbadensis* is recognized for its exceptional medicinal properties [5]. *Aloe vera* offers several advantages that make it suitable for various drug delivery systems. It can be easily extracted with minimal equipment, is cost-effective, readily available, and generally safe for use. Additionally, *Aloe vera* facilitates intestinal absorption, prolongs drug release from pharmaceutical dosage forms, and improves the efficient delivery of poorly absorbable drugs. In the pharmaceutical industry, *Aloe vera* finds applications in topical preparations such as gels and ointments, as well as in the production of directly compressible matrix tablets and capsules [6].

Hydrogels are three-dimensional polymeric networks composed of a polymer backbone, water, and a crosslinking agent. These networks have a hydrophilic nature, allowing them to absorb water or physiological fluids [7]. Hydrogels can be crosslinked either physically or chemically. This crosslinking mechanism enables the hydrogels to retain their structure even when they absorb significant amounts of water or biological fluids. Moreover, the crosslinking process facilitates the controlled release of active agents by immobilizing them within the hydrogel matrix [8,9,10]. Hydrogel-based drug delivery systems have gained significant interest in various applications such as dental treatments, injectable polymers, stimuli-responsive systems, topical applications, implants, and sustained-release drug-delivery systems [11]. These systems utilize hydrogels that can undergo changes in permeability, mechanical strength, and swelling behavior in response to various stimuli such as electric fields, magnetic fields, ionic strength, pH, and temperature [12]. Compared to conventional systems, hydrogels offer therapeutic benefits through prolonged and sustained drug action. They are also biocompatible and biodegradable, enabling site-specific drug delivery [13]. The key components of hydrogel formulations include polymers, monomers, crosslinkers, and initiators, all of which influence the swelling behavior of hydrogels. The applications of hydrogels primarily rely on the swelling behavior of the polymeric network. By modifying the formulation components, the swelling behavior of hydrogels can be altered to control and modify drug release. Several studies have indicated that the swelling properties of crosslinked hydrogels can be modified by altering the formulation components, including the concentration of the polymer, monomer, crosslinking agent, and initiator [14,15,16,17].

The objective of this study was to develop polymeric networks using *Aloe vera* mucilage through the free-radical polymerization method. The researchers aimed to investigate the influence of different concentrations of *Aloe vera* polymer, acrylamide monomer, and methylene bisacrylamide crosslinking agent on the swelling behavior and subsequent release of the model drug thiocolchicoside. The impact of these formulation parameters on drug release was assessed by evaluating important parameters such as swelling ratio, equilibrium swelling, porosity, and sol–gel fraction of the formulated polymeric networks. Several advanced characterization techniques including FTIR, XRD, SEM, TGA, and DSC were employed to thoroughly analyze the *Aloe vera*–*AAm* polymeric networks. Additionally, drug-release kinetic models were utilized to gain insights into the mechanism of drug release from the formulated polymeric networks.

## 2. Results and Discussion

**Figure 1:**
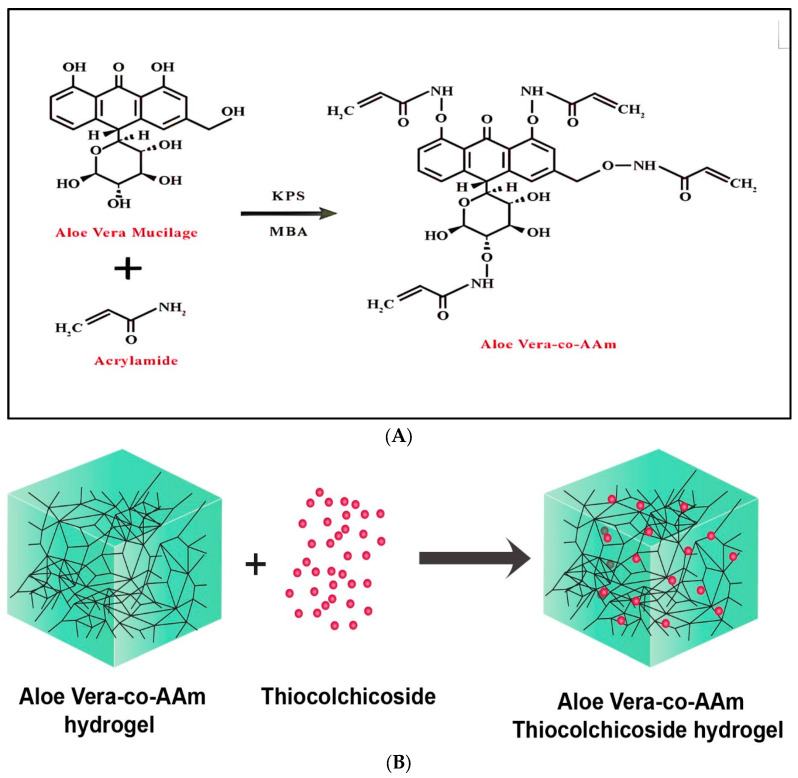
(**A**) Schematic diagram for the synthesis of *Aloe vera* crosslinked with acrylamide. (**B**). Structural diagram of the polymeric network.

The proposed chemical structure of *Aloe vera* crosslinked with acrylamide (*AAm*) is illustrated in Figure 1A while the structural diagram of the hydrogel network is illustrated in Figure 1B. As shown in Table 1, nine different formulations were articulated via a free=radical polymerization method, with varying concentrations of polymer (*Aloe vera*), crosslinker (Methylene bisacrylamide) and monomer (acrylamide).

### 2.1. Swelling Behavior of Aloe vera–AAm Polymeric Networks

**Figure 2:**
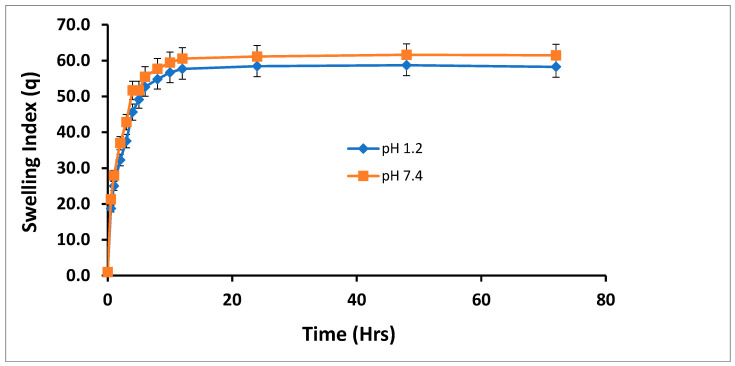
Swelling index of the *Aloe vera*–*AAm* polymeric network formulations at pH 1.2 and 7.4.

Three sets of polymeric networks were synthesized to evaluate the effect of polymer, monomer, and crosslinker on the swelling index. The swelling index of all *Aloe vera*–*AAm* polymeric network formulations were analyzed at pH 1.2 and 7.4 at different time intervals until equilibrium swelling was established, as shown in Figure 2. Since acrylamide is nonionic in nature, it does not carry any charge in solution. As a result, it does not exhibit significant response to changes in pH. This lack of charge-based interactions leads to a consistent swelling behavior of acrylamide-based materials, regardless of whether the pH is 1.2 or 7.4. However, the slight differences in swelling observed between pH 1.2 and 7.4 of acrylamide hydrogel could potentially be attributed to differences in salt concentrations between the buffers. At pH 1.2, typically used as a simulated gastric fluid, the buffer may contain higher concentrations of chloride ions (e.g., from hydrochloric acid). These chloride ions can contribute to a higher ionic strength, leading to increased electrostatic interactions within the hydrogel network. This enhanced ionic strength can promote stronger crosslinking or ionic bonding within the hydrogel, potentially resulting in reduced swelling. On the other hand, at pH 7.4, which corresponds to a more neutral physiological condition, the buffer may contain lower concentrations of chloride ions. This lower ionic strength may weaken the electrostatic interactions within the hydrogel network, allowing for increased swelling due to reduced crosslinking or ionic bonding [18,19].

### 2.2. Effect of Varying Polymer Concentration on the Aloe vera–AAm Polymeric Network

**Figure 3:**
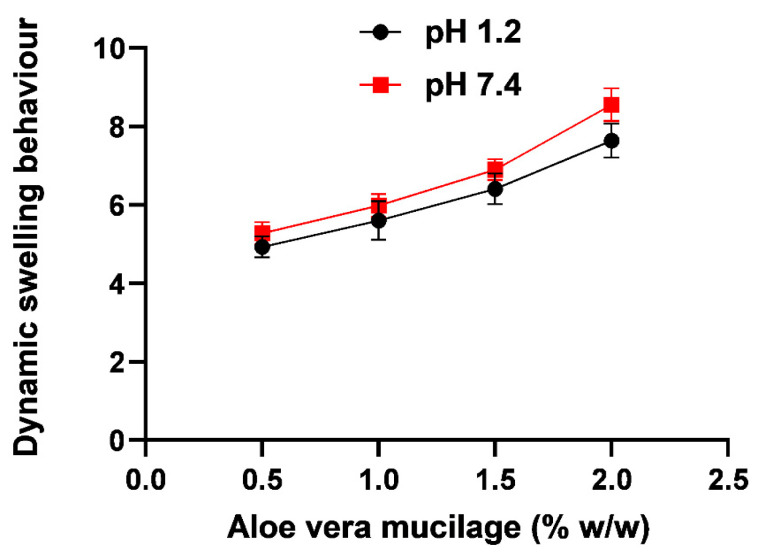
Dynamic swelling behavior of the *Aloe vera*–*AAm* polymeric networks with varying *Aloe vera* mucilage concentration at pH 1.2 and 7.4.

Swelling of naturally occurring polysaccharides depends upon the presence of hydrophobic/hydrophilic groups, degree of crosslinking, and elasticity of the network. Swelling ratios of all formulations with increasing polymer concentrations (0.5%, 1%, 1.5%, and 2%) at pH 1.2 and 7.4 were conducted. Results (Figure 3) indicated that swelling increased with an increase in polymer concentration. In the literature, most researchers have attributed an increase in swelling index with an increase in polymer content due to the hydrophilic nature of polysaccharides. Polymeric networks with hydrophilic groups show greater swelling than do polymeric networks containing hydrophobic groups because the swelling index of hydrogel decreases with the increasing ratio of hydrophobic polymer. An increased hydrophobic concentration in formulation decreases the polymer relaxation and reduces the water diffusion ability of hydrogel, thus minimizing their interaction with a water molecule [20,21]. The swelling of a polymer depends on factors such as the number of hydrophilic groups present in the polymeric networks and the crosslinking density of the polymer’s fine structure. When the concentration of the polymer increases, the concentration of hydrophilic groups also increases, leading to a lower crosslinking density. This, in turn, results in increased swelling of the polymer [22,23].

### 2.3. Effect of Varying the Monomer Concentration on the Aloe vera–AAm Polymeric Network

**Figure 4:**
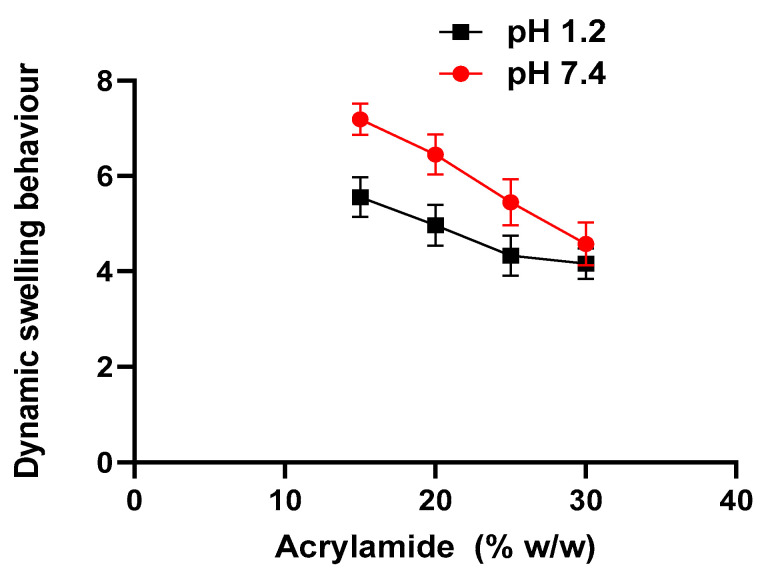
Dynamic swelling behavior of *Aloe vera*–*AAm* polymeric networks with varying monomer concentrations at pH 1.2 and 7.4.

Swelling ratios of all formulations of *Aloe vera*–*AAm* polymeric network with varying monomer concentrations of M1 and M2 at pH 1.2 and 7.4 were conducted, the results of which are shown in Figure 4. A decrease in swelling ratio was seen with increasing acrylamide content (15% and 20%). The decrease in swelling ratio with increasing acrylamide content (15% and 20%) can be attributed to factors such as higher cross-linking density, increased network rigidity, polymer chain interactions, and reduced water accessibility. These factors limit the mobility of polymer chains, restrict the penetration of solvent molecules, and decrease the hydrogel’s hydrophilicity, leading to reduced swelling [24,25,26,27]. An increase in monomer concentration leads to the evolution of heat increment during the reaction. This increase in monomer concentration induces a heat-induced auto cross-linking and branching reaction, resulting in a reduction in swelling capacity. Moreover, higher monomer concentration is associated with a shorter gelation time, as a higher kinetic chain length lowers the swelling capacity. Consequently, as the monomer content increases, the swelling capacity of copolymers decreases [27]. Furthermore, increasing monomer concentration leads to a decrease in swelling of the polymeric network. For a constant gel volume, a decrease in monomer concentration increases the degree of dilution of the matrix within the gel, subsequently increasing the equilibrium water content of the gel [28]. Conversely, a decrease in acrylamide content results in increased swelling due to reduced crosslinking and the increased microporosity of the polymeric network [29,30].

### 2.4. Effect of Varying Crosslinker Concentration on the Aloe vera–AAm Polymeric Network

**Figure 5:**
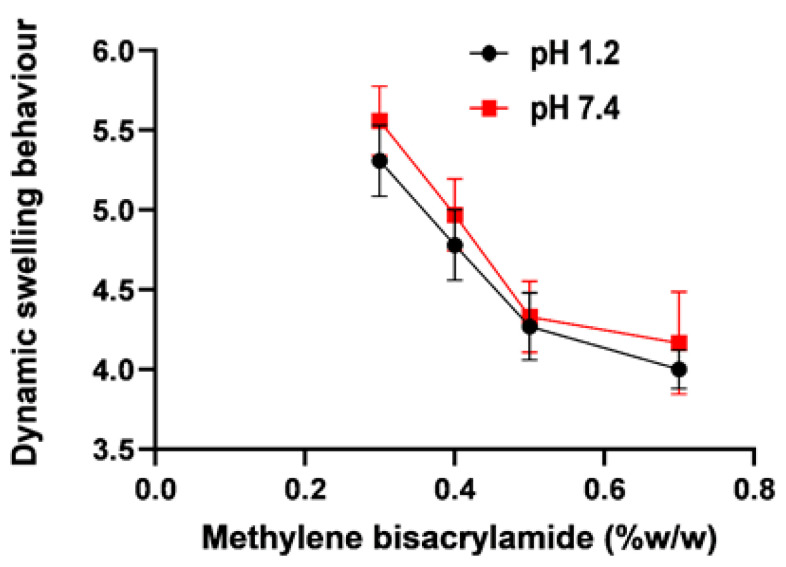
Dynamic swelling behavior of *Aloe vera*–*AAm* polymeric networks with varying cross linker concentrations at pH 1.2 and 7.4.

Swelling ratios of all formulations of the *Aloe vera*–*AAm* polymeric network with varying content of MBA (0.3%, 0.5%, and 0.7%) at pH 1.2 and 7.4 were conducted, the results of which are shown in Figure 5. It is evident from the results that increasing the crosslinker concentration decreases the swelling ratio and % equilibrium swelling, as the crosslinking agent concentration is directly related to the crosslinking density, which markedly affects the dynamic swelling ratio of the polymeric network. This was because there was an increase in the structural stability of the network and a decrease in network mesh size as the crosslinking agent contents were increased. The highly crosslinked rigid structure cannot not be expanded; as a result, low uptake of fluid by the polymer ultimately leads to a lower swelling ratio and equilibrium swelling [31]. Similar results were reported in which a less-porous network structure was produced with a high degree of crosslinking of the polymeric network that possessed a low swelling ratio [32].

### 2.5. Gel Content

**Figure 6:**
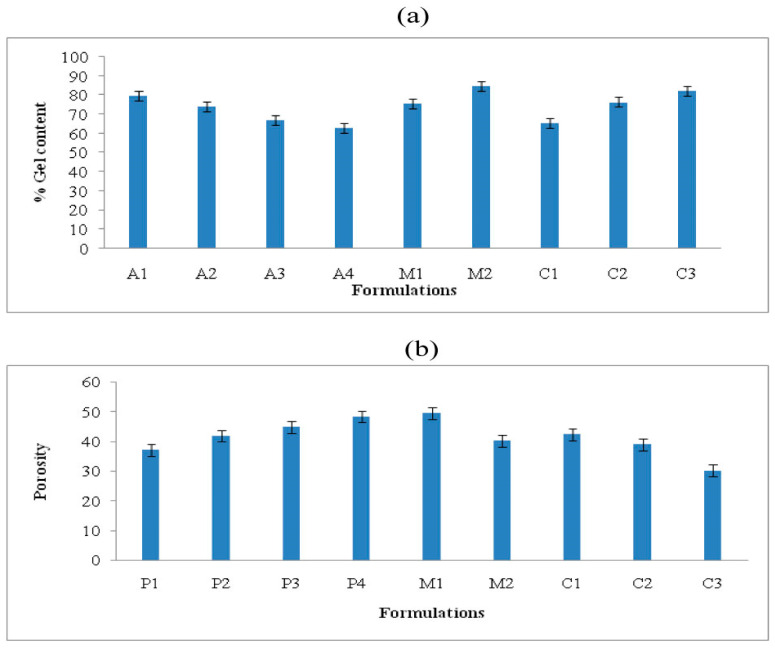
Percent gel content (**a**) and porosity measurement (**b**) of the *Aloe vera*–*AAm* polymeric network formulations.

The gel content of the *Aloe vera*–*AAm* polymeric network formulations was assessed, the results of which are presented in Figure 6a. The results showed that with an increase in the concentration of *Aloe vera* mucilage from 0.5% to 2%, the percent gel content decreased from 79.88% to 62.91%. This observation can be attributed to the fact that the gel content is influenced by the crosslinking density, which in turn affects the movement of free radicals during the polymerization process [33]. The decrease in gel content with higher polymer concentration results in a denser solution, limiting the escape of bubbles from the gel matrix and leading to a reduction in the crosslinking process [34].

On the other hand, when the monomer concentration was increased from 15% to 20%, and the crosslinking agent concentration was increased from 0.3% to 0.7%, the percent gel content increased from 75.43% to 84.53% and from 65.36% to 82.22%, respectively. This increase in gel content can be attributed to the higher concentration of monomer and crosslinker, which promotes more crosslinking and consequently enhances the gel strength [35,36].

### 2.6. Porosity

The porosity of the *Aloe vera*–*AAm* polymeric network formulations was evaluated, the results of which are presented in Figure 6b. Porosity is a measure of the pore volume within the polymeric networks. The results showed that increasing the concentration of *Aloe vera* mucilage led to an increase in porosity, ranging from 37.04% to 48.34%. This can be attributed to the higher viscosity of the solution with increased *Aloe vera* mucilage content. The higher viscosity impedes the involvement of free radicals and hinders the polymerization process. Consequently, the resulting polymeric network has interconnected channels, lower crosslinking density, and higher porosity [37].

In contrast, when the concentration of monomer and crosslinker was increased, the porosity of the polymeric network decreased. The porosity decreased from 49.33% to 40.12% with increasing monomer concentration and from 42.27% to 30.13% with increasing crosslinker concentration. This decrease in porosity can be attributed to the higher crosslinking density achieved with increased monomer and crosslinker concentrations. The increased crosslinking density leads to molecular entanglements, reducing the mesh size and pore volume of the polymeric network, resulting in decreased porosity. Similar findings have been reported by other researchers, highlighting the inverse relationship between crosslinking density and porosity [38].

### 2.7. Mechanical Strength

Young’s modulus (E) is known to increase in relation to the polymerization or crosslinking density of a material. This means that as the polymer chains become more tightly bonded through crosslinking, the mechanical strength of the material also increases. Factors that contribute to increased crosslinking density consequently lead to enhanced mechanical strength. One such factor is the concentration of monomers and crosslinkers. When the concentration of these components is increased, more crosslinking reactions occur, resulting in a higher density of crosslinks throughout the material. As a result, the gel strength of the material increases, leading to a corresponding increase in mechanical strength.

### 2.8. Loading of Drug

The amount of thiocolchicoside incorporated into the various formulations of the *Aloe vera*–*AAm* polymeric network is presented in Table 2. The drug-loading capacity was associated with the swelling behavior of the formulations. Among all the formulations, M1 exhibited the highest thiocolchicoside loading, with a calculated value of 35 mg using the weight method. This is consistent with the swelling profile of the M1 formulation, which showed the highest degree of swelling compared to the other formulations of the *Aloe vera*–*AAm* polymeric network. The increased swelling observed in the M1 formulation can be attributed to the presence of a larger number of pores within the polymer network. These pores facilitate the penetration of the drug solution into the swollen hydrogel, leading to a higher absorption of the drug solution. Consequently, the M1 formulation exhibits a higher drug-loading capacity within the hydrogel system [37].

These findings highlight the relationship between the swelling behavior of the polymeric network and the drug-loading capacity. Formulations with greater swelling capabilities tend to have higher drug absorption and loading capacities, making them potential candidates for efficient drug delivery systems.

### 2.9. Fourier Transform Infrared Spectroscopy

**Figure 7:**
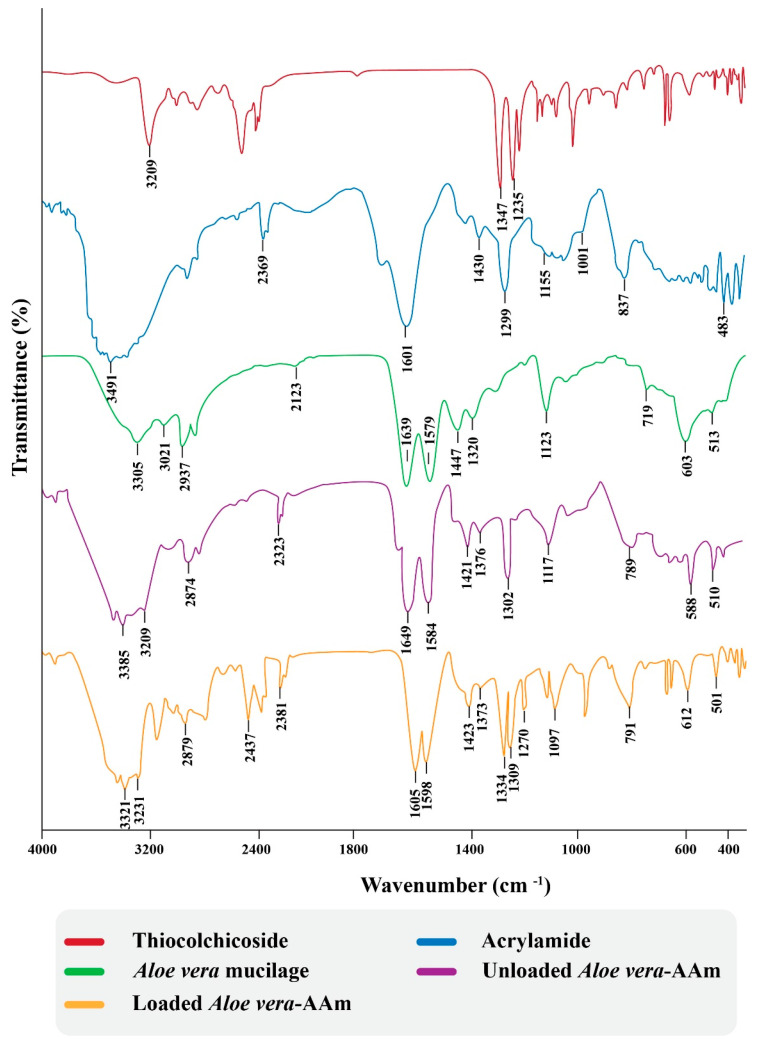
FTIR of individual components and the unloaded and loaded polymeric network.

An FTIR spectrometer is one of the fascinating tools for elucidating some structural features of organic compounds. A FTIR spectrometer can provide valuable information regarding functional groups. FTIR spectra of pure *Aloe vera* mucilage, acrylamide, thiocolchicoside, and the unloaded and loaded *Aloe vera*–*AAm* polymeric network were recorded using KBr pellet disc methods, as shown in Figure 7. A spectrum of *Aloe vera* mucilage showed absorption at 3021 and 3305/cm for OH-stretching vibration of uronic acid, mannose, and galacturonic acid, and phenolic groups in the antraquinones present in AV, whereas 1639/cm was used for C=O stretching. Besides these characteristic peaks, some other prominent peaks were also observed at 1123, 1320, 1579, and 2937/cm corresponding to the C-O-C stretches of acetyl groups, which may indicate the presence of storage bioactive polysaccharides, such as acemannans and glucamannans [38,39]. In case of spectrum of acrylamide, the characteristic peaks at 3491/cm highlight symmetric and asymmetric stretching of NH-stretching while the C=O characteristic vibrational bands of amide were observed at 1601/cm. Some other prominent peaks at 1430, 1299, 1155, 1001, 837, and 483/cm were representative of CH2-deformed, CN-stretching, NH2-rocking, CH2-wagging, C=O wagging, NH2-twisting, and C-CN deformation, respectively [40]. FTIR-spectrum of the *Aloe vera*–*AAm* polymeric network showed some additional peaks, potentially confirming the graft copolymerization in between polymer (*Aloe vera*) and monomer (acrylamide). A characteristic peak at1649, 1584/cm was due to the C=O stretching amide. Meanwhile, some peaks at 1376, 1302, and 1117/cm (CN-stretching) and at 3305 and 3021/cm (NH-stretching) provided enough evidence for the possible copolymerization between polymer and monomer [41]. The FTIR-spectrum of pure thiocolchicoside showed characteristic peaks at 3209/cm (OH-stretching) and thioether bands at 2511/cm, 1343/cm (carbonyl tertiary amide), 1235/cm (CN-stretching), and 1051 (C-OH stretching) [39]. In the FTIR-spectrum of THC-loaded *Aloe vera*–*AAm* polymeric network, some characteristics peaks were overlapped with a slight shift of band which indicated the absence of any interaction between drug and monomer during their grafting [42]

### 2.10. Scanning Electron Microscopy

**Figure 8:**
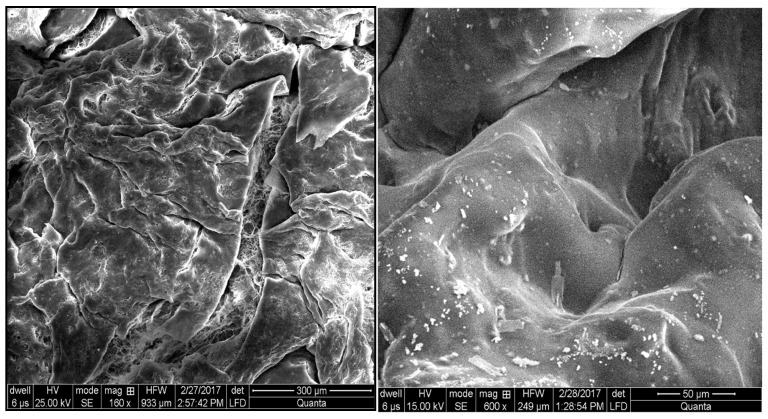
SEM images of the loaded polymeric network.

The results of scanning electron microscopy (SEM) analysis of the lyophilized *Aloe vera*–*AAm* polymeric network are depicted in Figure 8. The SEM images confirm that the synthesized polymeric network exhibits an irregular and porous fibrillar structure. These pores within the network play a crucial role in facilitating the passage of water or other fluids. Furthermore, these pores also serve as the sites where the hydrophilic groups of the polymeric network interact with external stimuli. The presence of this porous structure on the surface of the polymeric network supports its high swelling tendency. The interconnected pores provide ample space for the absorption and retention of water or other fluid environments. This characteristic is essential for hydrogels intended for drug delivery applications, as it allows for efficient diffusion of drugs and promotes sustained release. The SEM images provide visual evidence of the porous and interconnected structure of the *Aloe vera*–*AAm* polymeric network, confirming its potential as a suitable material for applications such as drug delivery systems and other biomedical applications.

### 2.11. Thermogravimetric Analysis (TGA) and Differential Calorimeter (DSC)

**Figure 9:**
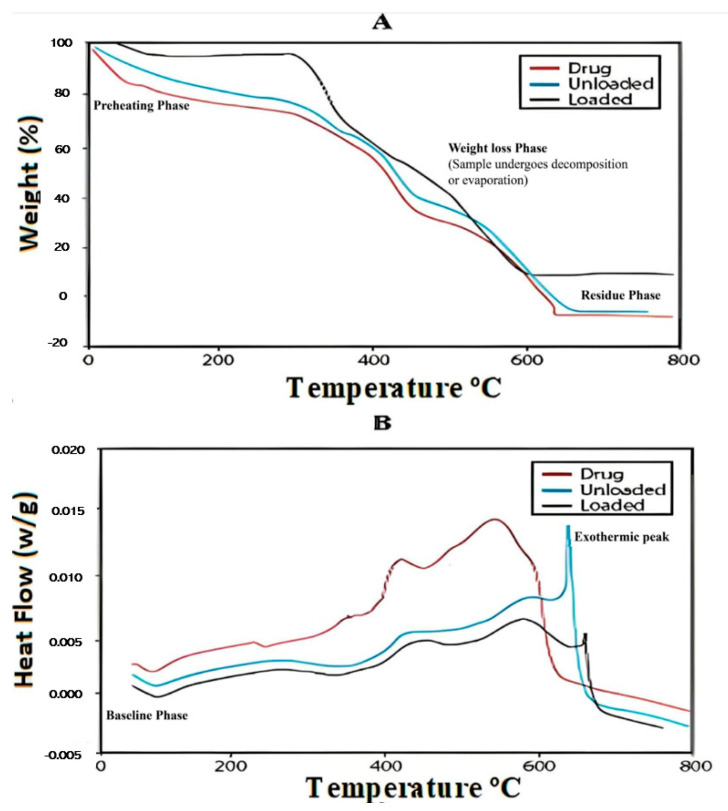
TGA and DSC curve of drug, drug unloaded, and loaded polymeric network.

Thermogravimetric analysis (TGA) and differential scanning calorimetry (DSC) of the pure drug, loaded and unloaded *Aloe vera*–*AAm* polymeric network was performed to identify the thermal stability of prepared hydrogel formulation and drug. The overlay of the TGA analysis is presented in Figure 9 A. It was evident from the TGA curve that THC showed a 17% weight loss at 277 °C. At the same temperature, the THC-loaded *Aloe vera*–*AAm* polymeric network showed a 5% weight loss. At 580 °C, the drug showed 91% weight loss while at this stage, the *Aloe vera*–*AAm* polymeric network showed 84% weight loss. These results demonstrated that the thermal stability of the loaded polymeric network increased due to the incorporation of thiocolchicoside [43].

Differential scanning calorimetry (DSC) analysis was conducted to verify the identity of the drug in its pure form and in the drug-loaded polymeric formulations. Figure 9B presents an overlay of the DSC thermograms of the pure drug, drug-loaded polymeric networks, and unloaded polymeric networks. The pure thiocolchicoside exhibited a distinct sharp endothermic peak at 213 °C, which corresponds to its melting point. However, in the drug-loaded polymeric networks, the characteristic melting peak of thiocolchicoside was not clearly observed. This suggests that the drug was either molecularly dispersed within the polymer or existed in an amorphous form. These observations indicate that the drug and polymer were compatible with each other, as the drug did not undergo any significant thermal interactions that would alter its crystalline structure. The DSC analysis confirms the successful incorporation of thiocolchicoside into the polymeric networks and suggests that the drug was well-dispersed within the polymer matrix, which is favorable for its controlled release from the formulation [44].

### 2.12. X-ray Diffraction Analysis

**Figure 10:**
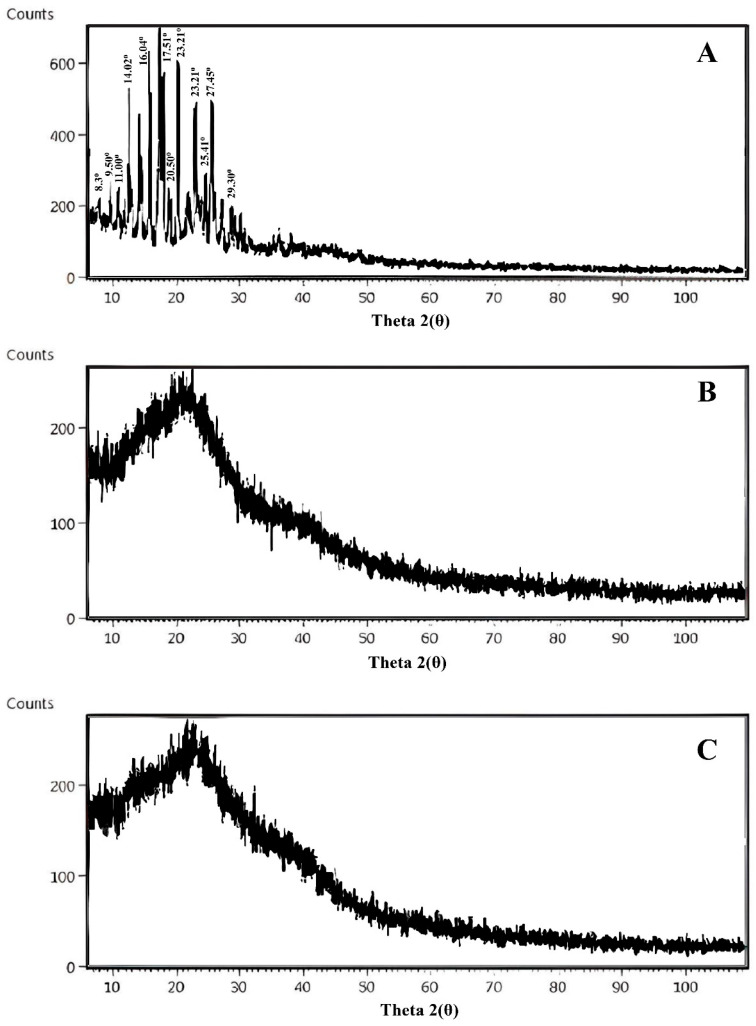
XRD spectra of (**A**) drug (**B**) unloaded polymeric network. (**C**) Loaded polymeric network.

XRD is a useful technique and can be used as an identification tool for the determination of the crystalline or amorphous nature of drugs. Distinct peaks in the XRD pattern of the pure drug were scattered at 2θ = 8.3°, 9.50°, 11.00°, 14.02°, 16.14°, 17.51°, 20.50°, 23.21°, 25.41°, 27.45°, and 29.30° (Figure 10), confirming its crystalline nature [45]. The XRD pattern of the drug-loaded polymeric network did not exhibit any characteristic peaks corresponding to thiocolchicoside. Instead, the diffraction pattern closely resembled that of the *Aloe ver-AAm* polymeric network. These findings suggest that thiocolchicoside underwent a transformation from a crystalline state to an amorphous state within the polymeric matrix. The absence of characteristic peaks indicates that the drug molecules were evenly dispersed within the polymer at the molecular level, resulting in the loss of their crystalline structure.

Similar results have been reported in previous studies, further supporting the notion that the drug–polymer interaction led to the amorphization of thiocolchicoside within the polymeric network [46,47]. This transformation to an amorphous state is favorable for the controlled release of the drug from the polymeric formulation.

### 2.13. Method Validation

**Figure 11:**
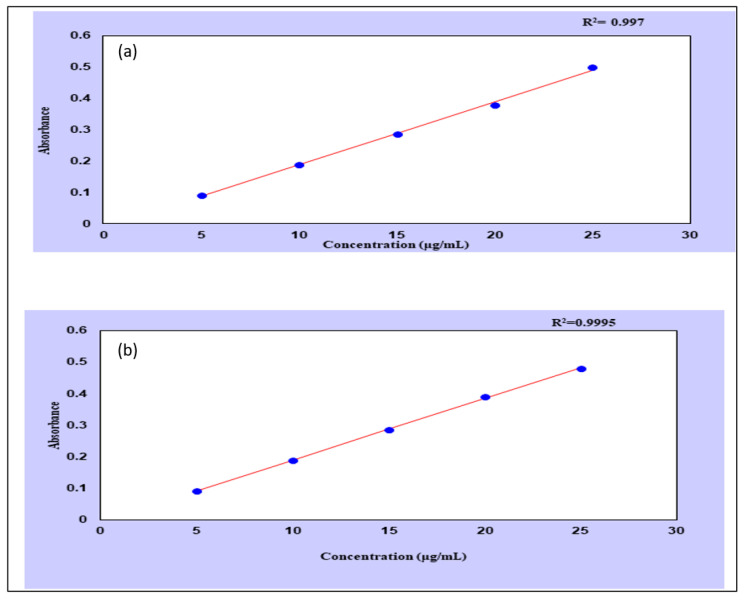
Calibration curve of thiocolchicoside at pH 1.2 (**a**) and 7.4 (**b**).

A UV spectrophotometer was used to draw standard curves of thiocolchicoside at pH 1.2 (Figure 11a) and pH 7.4 (Figure 11b). The wavelength corresponding to the maximum absorbance in pH 1.2 and pH 7.4 was found to be 259 nm. The method was validated according to ICH guidelines for the determination of thiocolchicoside at pH 7.4, as shown in Table 3. Beer’s law was obeyed over the concentration range of 5–25 µg mL^−1^ with a correlation coefficient (r^2^) value of 0.9995. The limit of detection (LOD) and limit of quantification (LOQ) were 0.1326 and 0.4019 µg mL^−1^, respectively, indicating the sensitivity of the method. The percent recovery values ranged from 99.53 to 99.76% indicating the accuracy of the method (Table 4). The method was also found to be precise and robust, as the %RSD values were less than 2% (Table 5, Table 6 and Table 7).

### 2.14. In Vitro Release Profile of the Aloe vera–AAm Polymeric Network Formulations

It was observed that the release behavior of all formulations of the *Aloe vera*–*AAm* polymeric network did not exhibit pH-dependent release, which is similar to the swelling behavior observed at pH 1.2 and pH 7.4. This can be attributed to the nonionic nature of the monomer (acrylamide) [48]. The release of thiocolchicoside from the polymeric network is influenced by factors such as swelling, drug–polymer interactions, and drug solubility [49]. Figure 11 demonstrates that as the concentration of *Aloe vera* mucilage increased from 0.5% to 2% (A1 to A4), the release of thiocolchicoside from the polymeric network increased. The formulation A4 (2%) exhibited the highest release, reaching 85.18% at pH 1.2 and 86.57% at pH 7.4. This increase in release can be attributed to the presence of more hydrophilic groups. With an increase in polymer content, the hydrogel becomes highly hydrated due to the availability of more hydroxyl groups, resulting in enhanced drug release [50,51].

Furthermore, among the formulations with varied acrylamide concentrations (M1 to M2), the percentage of thiocolchicoside release decreased with increasing acrylamide concentration. The highest release was observed in M1 (15% acrylamide concentration), which reached 88.84% at pH 1.2 and 89.57% at pH 7.4. This can be attributed to the fact that increasing the acrylamide content in the hydrogel led to an increase in crosslinking density and a decrease in microporosity. Additionally, hydrophilicity decreased due to the involvement of hydrophilic groups in hydrogen bonding between the hydrophilic groups of *Aloe vera* mucilage and the amide group of acrylamide. As a result, the swelling decreased, leading to a lower percentage of drug release [52,53].

**Figure 12:**
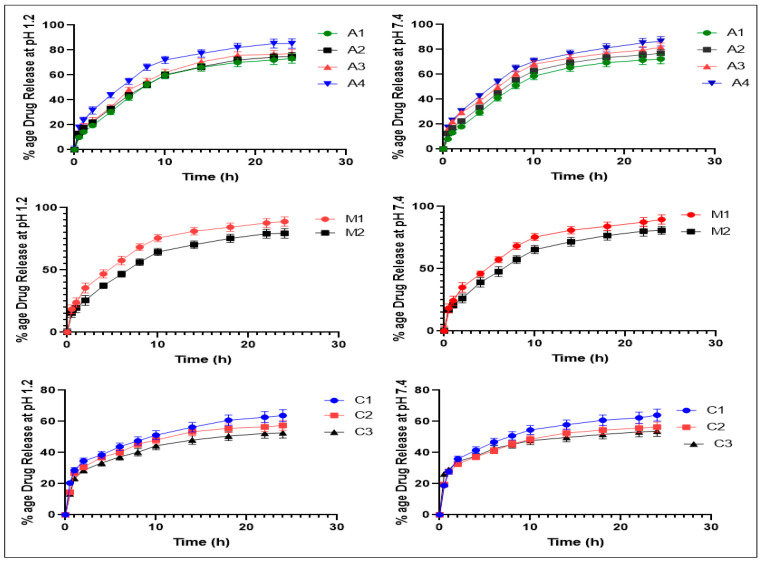
Percentage drug release of the *Aloe vera*–*AAm* polymeric network formulations at pH 1.2 and 7.4.

Figure 12 presents the comparison between formulations C1, C2, and C3, which contain MBA concentrations of 0.3%, 0.5%, and 0.7%, respectively. The highest release was observed in the formulation with the lowest concentration of MBA (C1), which reached 63.78% at pH 1.2 and 64.01% at pH 7.4. This can be attributed to the fact that a more crosslinked network is formed with increasing crosslinker concentration. This behavior of the network leads to a decrease in chain relaxation and subsequently reduces drug release. Therefore, the release of the drug is more challenging from a highly crosslinked copolymer compared to a polymer with lower crosslinking density [54].

### 2.15. Drug Release Kinetics

The release data were fitted to various release models (zero order, first order, Higuchi, Korsmeyer–Peppas, and Hixson–Crowell) to evaluate the release kinetics of thiocolchicoside. The model that best fit the release data was evaluated using the regression coefficient (R^2^). Values of R^2^ for all models obtained from the drug-loaded *Aloe vera*–*AAm* polymeric network at varying content of the polymer, monomer, and crosslinker at pH 1.2 and 7.4 are given in Table 8. Based on the highest regression coefficient value (R^2^), the best-fit model found was to be Korsmeyer–Peppas. The Korsmeyer–Peppas model is used to describe drug release from the polymeric system and is applicable when the release mechanism is unknown or more than one type of phenomenon is involved. It considers that often drug release deviates from Fick’s law and follows an anomalous behavior [55]. Release kinetics in the swellable system is governed by a rate of liquid diffusion and the rate of polymer chain relaxation. When the rate of liquid diffusion is slower than is the rate of polymer chain relaxation, the mechanism of release is Fickian. When the relaxation process is slower than the liquid diffusion rate, case II transport occurs. However, when both processes occur at the same magnitude, the release becomes non-Fickian or anomalous. The values of release exponent (n) are also given in Table 8. According to the value of the release exponent, the drug release mechanism from the *Aloe vera*–*AAm* polymeric network was Fickian, i.e., rate of liquid diffusion was slower than was the rate of polymer chain relaxation. Gentamicin release from poly (acrylic acid) and gelatin hydrogel also follows Fickian diffusion [56].

## 3. Conclusions

This study, utilized *Aloe vera* mucilage, a natural polymer, to create a different polymeric formulation by varying the amounts of the polymer, monomer, and crosslinker. Among all formulations, M1 exhibited excellent properties in terms of swelling and sustained release of the drug. It offered the advantage of prolonged drug release, which resulted in a reduced frequency of dosing. This is beneficial for patients, as it improves compliance with the medication regimen. Hence, the use of *Aloe vera*-based polymeric networks proved to be a promising approach for achieving sustained drug delivery. The findings suggest that this natural polymer can be utilized as a suitable carrier system, providing the potential for enhanced therapeutic outcomes and improved patient convenience. While the current study focuses on the characterization and performance evaluation of the hydrogel system with thiocolchicoside as a model drug, the incorporation of different nanocontrolled systems holds promise for expanding the functionality and therapeutic applications of the hydrogel platform.

## 4. Materials and Methods

### 4.1. Materials

*Aloe vera* was purchased from the local food market of Sargodha, Pakistan. Acrylamide, sodium hydroxide, and potassium dihydrogen phosphate were from Sigma Aldrich, Germany. N, N-Methylene bis-acrylamide was obtained from Fluka, Buchs, Switzerland. Hydrochloric acid and absolute ethanol were acquired from Riedel-de Haen, Germany. Thiocolchicoside was obtained as a gift sample from Pharmevo (Pvt) Ltd., Karachi, Pakistan.

### 4.2. Methods

#### 4.2.1. Extraction of *Aloe vera* Mucilage

The water exaction method was used, as it includes the cleaning, homogenization, separation, and centrifugation of *Aloe vera*. The supernatant was collected and mixed with absolute ethanol at a ratio of 1:3 for 12 h. Mucilage was collected, washed with a sufficient quantity of n-hexane, and dried in an oven at 50 °C for 3–4 days. Finally, dried *Aloe vera* mucilage was ground to a fine powder and stored in airtight jars in a desiccator under a vacuum [57].

#### 4.2.2. Preparation of the *Aloe vera*–*AAm* Polymeric Network

Polymeric networks of *Aloe vera* were synthesized using the free-radical polymerization method, with varying concentrations of polymer extracted from *Aloe vera*, monomer (acrylamide), and crosslinker (methylene bisacrylamide) being incorporated. A measured amount of *Aloe vera* mucilage was suspended in distilled water, and the mixture was stirred using a magnetic stirrer on a hot plate at 70 °C. Potassium persulfate (KPS) was added as an initiator, and the mixture was stirred for an additional 10 min to generate free radicals. The reaction mixture was then cooled to room temperature. In a separate solution, acrylamide (monomer) and methylene bisacrylamide (crosslinking agent) were prepared and added to the reaction mixture under magnetic stirring at room temperature. The weight makeup process was carried out by adding distilled water. The reaction mixture was transferred to a test tube, which was placed in a water bath for polymerization. The temperature was gradually increased to 80 °C. After the reaction was complete, the obtained copolymers were removed from the water bath and allowed to cool to room temperature. The formulated polymeric networks were then cut into discs of 5 mm thickness using a sharp cutter. The discs were washed with a solution of ethanol and water (30:70) to remove any untreated monomers and excess reagents. After 24 h, the ethanol and water solution were replaced with a fresh solution for an additional 24 h. The washed discs were dried at 50 °C until a constant weight was achieved and then stored in air-tight containers for further characterization [58,59,60].

### 4.3. Swelling Studies

Swelling studies of all formulations were conducted at pH 1.2 and 7.4 to optimize and determine pH sensitivity. Dried discs were weighed and immersed in 100 mL of USP buffer solutions with pH 1.2 or pH 7.4 at 37 °C. Swollen samples were removed from buffer solutions after regular time intervals and weighed; excess surface water was removed by blotting using laboratory filter paper before weighing. The swelling index (q) was calculated using the following equation:

q = Ws/Wd (1)

where ‘q’ is the dynamic swelling index, ‘Ws’ is the swollen gel’s weight at time t, and ‘Wd’ is the initial weight of the sample at t_0_ [61,62].

### 4.4. Percentage Gel Content Determination

Fresh discs of all preparations that were not previously washed were dried in an oven at 50 °C until a constant weight (W_0_) was achieved. Then, dried discs of the polymeric network were dipped in distilled water for 72 h at room temperature to remove unreacted monomer and were again dried to a constant weight (W_1_) in an oven at 50 °C [63,64]. The gel content was then measured as follows:

Gel fraction (%) = (W_1_/W_0_) × 100 (2)

where ‘W_o_’ is the weight of the dried disc before extraction, and ‘W_1_′ is the weight of the dried disc after extraction.

### 4.5. Porosity Measurement

For porosity calculation, the solvent replacement method was used. Previously dried discs of the polymeric network were first weighed (M_1_) and then immersed overnight in absolute ethanol. After that, a laboratory filter paper was used to clear excess ethanol from the discs and was weighed again (M_2_). The porosity was calculated by following the equation:

Porosity = (M_2_ − M_1_)/ρV × 100 (3)

where ‘M_1_′ is the mass of dried disc before immersion, ‘M_2_′ is the mass of disc after immersion in absolute ethanol, ‘ρ’ is the density of absolute ethanol, and ‘V’ is the volume of hydrogel [65].

### 4.6. Mechanical Strength Determination

Evaluation of mechanical properties of produced hydrogels was carried out by using a static compression test, based on which the Young’s modulus values were determined. Mechanical strength of hydrogels as fully swollen hydrogels was determined by applying the weight on them until the hydrogels were fractured. All samples were tested under physiological conditions, i.e., 37 °C in PBS. Sample dimensions were measured with calipers to calculate the cross-sectional area and applied stress. Young’s modulus was derived from the initial linear range of the stress–strain curve [66].

Young’s modulus is equal to the longitudinal stress divided by the strain, as follows:

(4) E=σ/∈

Equation (4) represents the relationship between Young’s modulus (E), longitudinal stress (σ), and strain (ε) [67].

### 4.7. Drug Loading

Water-soluble thiocolchicoside was used as a model drug to load into formulated discs. A 1% (*w*/*v*) solution of thiocolchicoside in distilled water was prepared. One disc of each formulation was dipped in 100 mL of 1% drug solution until swelling equilibrium occurred at room temperature. Discs were removed from the solution of thiocolchicoside and washed out with distilled water to remove any excess amount of drug. Then, oven-drying was applied at 50 °C until a constant weight was achieved [68,69,70].

The following formula was used to determine drug loading in polymeric networks:

Total drug loaded = W_L_ − W_U_ (5)

where, W_L_ is the weight of the dried drug-loaded disc, and W_U_ is the weight of the dried unloaded disc.

### 4.8. Characterization of the Polymeric Networks

#### 4.8.1. Fourier Transform Infrared (FTIR) Analysis

For confirmation of grafting and drug compatibility with polymeric network components, an FTIR spectrophotometer (IR prestige-21 Shimadzu, Singapore) was used. Pestle and mortar were used to crush samples. The powdered sample (20 mg) was mixed with potassium bromide (4 mg) and then compressed into a 12 mm semitransparent disc by applying a pressure of 60 kN (Pressure gauge, Shimadzu, Singapore) for 2 min. The FTIR spectrum was recorded using an FTIR spectrometer, covering the wavelength range of 4000–400 cm^−1^ [71].

#### 4.8.2. Scanning Electron Microscopy (SEM) Analysis

SEM analysis was utilized for visualizing the surface morphology of polymeric network discs [30]. Before the SEM analysis, the swollen sample was lyophilized. For lyophilization, the sample was cooled rapidly (105 K/s) to liquid nitrogen temperature to transform the water content into amorphous ice. After the cooling step, the sample was kept below the glass transition temperature of the water. To acquire a fresh surface, the sample was sublimated at −90°C and then imaged at (−130 to −140 °C) with an incident electron beam. The voltage range used for the cryo-SEM was 5 kV [72].

#### 4.8.3. Thermal Analysis (TGA and DSC)

The TGA (thermogravimetric analysis) and DSC (differential scanning calorimetric analysis were used to determine the rate of thermal decomposition and to confirm the thermal stability of the polymeric networks and drug. Dried and ground samples of pure drug, loaded and unloaded, were heated by placing a stainless-steel pan and scanned under a nitrogen atmosphere. The temperature range was from ambient temperature to 800 °C, and the scanning rate was 10 °C/min [73].

#### 4.8.4. X-ray Diffraction Analysis (XRD)

X-ray diffraction analysis was conducted to determine the change in the physical form of pure drug and loaded and unloaded drug polymeric networks with an X-ray diffractometer (Panalytical, Kassel, Germany). Copper kα radiation (λ = 0.154 nm) was generated at an angle between 0 and 50° (2θ) under a fixed-time mode with a step interval of 0.02° [74].

### 4.9. Method Validation

The method was validated according to the international conference on harmonization (ICH) guidelines on the Validation of Analytical Procedures [75,76,77,78,79].

#### 4.9.1. Calibration Curve of Thiocolchicoside

A standard calibration curve for carrying out thiocolchicoside release studies was drawn both for pH 1.2 HCl buffer and pH 7.4 phosphate buffers. Absorbance was measured at 259 nm in a UV Spectrophotometer, and a standard calibration curve was drawn.

#### 4.9.2. Linearity and Range

A series of dilutions (5, 10, 15, 20, and 25 µg/mL) was prepared from a stock solution to establish linearity and range. Concentration was plotted against absorbance to obtain a calibration curve, and a regression equation was determined. Slope, intercept, and the correlation coefficient were calculated using the least-squares method.

#### 4.9.3. Detection and Quantification Limits

Limit of detection (LOD) and limit of quantification (LOQ) were calculated as per the ICH guidelines using the standard deviation of the response and slope as follows:

LOD = 3.3 σ/S(6)

LOQ = 10 σ/S(7)

where ‘σ’ denotes the standard deviation of the Y-intercept of the regression line, and ‘S’ is the slope of the calibration curve.

#### 4.9.4. Accuracy

The accuracy of the method was evaluated by carrying out recovery studies. Known quantities of standard thiocolchicoside (80%, 100%, and 120%) were added to a preanalyzed thiocolchicoside solution (10 µg/mL) and analyzed in triplicate. The % recovery and % RSD values were calculated.

#### 4.9.5. Precision

Repeatability and intraday and interday variations were studied to ascertain precision. For repeatability studies, the absorbance of thiocolchicoside solution (15 µg/mL) was measured six times. For intraday variations, three replicate measurements at three different concentration levels (5, 15, and 25 µg/mL) within the linearity range were taken within one day. Similarly, for interday variations, three replicate measurements of the same concentrations were analyzed for three days consecutively. The % RSD was calculated in all cases.

#### 4.9.6. Robustness

As per the ICH guidelines, the robustness of the proposed method using a UV spectrophotometer for thiocolchicoside was determined by varying the wavelength for a 20 µg/mL standard solution, and the percentage relative standard deviation was calculated.

### 4.10. In Vitro Drug Release Measurement and Drug Release Kinetics

The in vitro dissolution studies were performed using a USP dissolution apparatus II at 37 ± 0.5 °C to evaluate the release behavior of loaded discs with thiocolchicoside. Loaded discs were submerged in 500 mL of dissolution medium of pH 1.2 and 7.4 separately at a 50 rpm stirring rate for maintaining a uniform drug concentration in a medium for 24 h. A 5 mL aliquot was taken out at distinct time intervals (0, 0.5, 1, 2, 4, 6, 8, 10, 14, 18, 22, and 24 h) and replaced with the same volume of fresh buffer solution. Thiocolchicoside release was determined by using a UV spectrophotometer at 259 nm [78,79,80]. Percent of drug release was determined by using the following equation:

% drug release = F_t_/F _load_ × 100 (8)

where F_t_ is the release of a drug at time t, and F_load_ is the amount of drug loaded in a disc.

The model-dependent approach was used to determine the thiocolchicoside release kinetics, and different kinetic models, including zero order, first order, Korsmeyer–Peppas, Higuchi, and Hixson–Crowell, were applied to interpret the release mechanism with DD solver. The equations used for these models were as follows:

Zero-order kinetics: Q_t_ = K_o_t (9)

First-order kinetics: ln (100-Q_t_) = ln100 − K_1_t (10)

Higuchi model: Q_t_ = K_H_ t^1^/_2_ (11)

Hixon–Crowell model: (100 − Q_t_)^1/2^ = 100^1/3^ − k_HC_ t (12)

where Q_t_ represents the %age of drug released at time t; and K_o_, K_1_, K_H_, and K_HC_ represent the zero-order, first-order, Higuchi, and Hixon–Crowell release rate constants, respectively.

Korsmeyer–Peppas model: Q_t_/Q_e_ = k_KP_ t^n^ (13)

where Q_t_/Q_e_ indicates the fraction of drug released at time t, k_KP_ is the release rate constant, and n is the release exponent. When the value of n is 0.45, the order of release is Fickian. When n = 1, it corresponds to case II transport. The mechanism of diffusion is non-Fickian when 0.45 < n < 1 [81].

## Figures and Tables

**Table 1:** Composition of the *Aloe vera*–*AAm* polymeric network.

Formulation Code	*Aloe vera* Mucilage Extract %	Acrylamide %	Initiator %	Crosslinker %
A1	0.5	25	0.4	0.4
A2	1	25	0.4	0.4
A3	1.5	25	0.4	0.4
A4	2	25	0.4	0.4
M1	1.5	15	0.4	0.4
M2	1.5	20	0.4	0.4
C1	1.5	20	0.4	0.3
C2	1.5	20	0.4	0.5
C3	1.5	20	0.4	0.7

**Table 2:** Drug loading in all formulations of the *Aloe vera*–*AAm* polymeric networks.

Formulation Code	Thiocolchicoside-Loaded mg/g Disk
	By Weight Method Mean ± * S.E.M
A1	19 ± 0.8
A2	23 ± 0.7
A3	30 ±1.4
A4	35 ± 0.5
M1	40 ± 0.7
M2	32 ± 1.6
C1	25 ± 0.9
C2	22 ± 1.4
C3	18 ± 1.2

* S.E.M = standard error of the mean

**Table 3:** Regression and analytical parameters.

Parameter	Value
λ max (nm)	259
Beer’s law limit (µg/mL)	5–25
Regression equation	y = 0.038x + 0.0233
Intercept σ	0.0233
Slope, S	0.038
Correlation coefficient (r^2^)	0.9995
Limit of detection (µg/mL)	0.1326
Limit of quantification (µg/mL))	0.4019

**Table 4:** Accuracy determination studies of thiocolchicoside.

% Drug Added	Amount of THC (µg) Added to 10 µg/mL THC Solution	The Total Amount of THC	Amount of THC Found (µg/mL)	% Recovery	Mean ± SD	% RSD
80	8	18	17.94	99.67	99.76 ± 0.15	0.12
80	8	18	17.95	99.72		
80	8	18	17.98	99.89		
100	10	20	19.87	99.35	99.53 ± 0.176	0.18
100	10	20	19.91	99.55		
100	10	20	19.94	99.7		
120	12	22	21.86	99.36	99.74 ± 0.366	0.37
120	12	22	21.95	99.77		
120	12	22	22.02	100.09		

**Table 5:** Repeatability studies of thiocolchicoside.

Concentration (µg/mL)	Absorbance	Mean ± SD	% RSD
15	0.2858	0.2858 ± 0.001	0.34
15	0.2857		
15	0.2859		
15	0.2856		
15	0.2858		
15	0.2859		

**Table 6:** Intraday precision and interday precision.

Intraday Precision of Three Different Concentrations of Thiocolchicoside					
Concentration (µg/mL)	Time				% RSD
	9:00 a.m.		11:30 a.m.	3:00 p.m.	
5	0.0921		0.0920	0.0922	0.108
15	0.2858		0.2856	0.2854	0.350
25	0.4795		0.4793	0.4791	0.208
Interday Precision of Three Different Concentrations of Thiocolchicoside					
**Concentration** **(µg/mL)**		**Day**			**% RSD**
		**1**	**2**	**3**	
5		0.0921	0.0929	0.0931	1.078
15		0.2858	0.2853	0.2851	0.500
25		0.4795	0.4789	0.4685	0.210

**Table 7:** Robustness study of thiocolchicoside.

Wavelength (nm)	Concentration (µg/mL)	Absorbance (Mean ± SD)	% RSD
258	20	0.3901 ± 0.002	0.512
259	20	0.3903 ± 0.001	0.256
260	20	0.3899 ± 0.001	0.257

**Table 8:** Release kinetics of thiocolchicoside from polymeric networks at pH 1.2 and pH 7.4.

Release Models											
Formulation Code	Zero Order		First Order		Higuchi		Korsmeyer–Peppas			Hixson–Crowell	
	K_0_	R^2^	K_1_	R^2^	K_H_	R^2^	k_KP_	R^2^	n	k_HC_	R^2^
*Aloe vera*–*AAm* polymeric networks at pH 1.2											
A1	3.87	0.685	0.08	0.941	16.35	0.971	17.59	0.953	0.45	0.02	0.888
A2	4.1	0.605	0.09	0.926	17.4	0.965	20.73	0.974	0.43	0.02	0.865
A3	4.34	0.518	0.11	0.915	18.56	0.96	24.31	0.984	0.4	0.03	0.847
A4	4.64	0.477	0.13	0.935	19.89	0.951	26.88	0.983	0.39	0.04	0.877
M1	4.82	0.435	0.14	0.943	20.7	0.941	28.83	0.982	0.37	0.04	0.893
M2	4.21	0.593	0.1	0.929	17.91	0.974	21.92	0.986	0.42	0.03	0.868
C1	3.47	0.545	0.06	0.839	14.81	0.965	18.9	0.984	0.41	0.02	0.764
C2	2.95	0.543	0.04	0.784	12.58	0.964	16.1	0.984	0.41	0.01	0.716
C3	0.56	0.594	0.05	0.781	10.9	0.971	13.28	0.983	0.43	0.01	0.726
*Aloe vera*–*AAm* polymeric networks at pH 7.4											
A1	3.82	0.713	0.07	0.948	16.09	0.971	16.56	0.971	0.45	0.02	0.9
A2	4.08	0.638	0.09	0.938	17.28	0.972	19.89	0.977	0.45	0.02	0.881
A3	4.34	0.52	0.11	0.916	18.56	0.958	24.22	0.982	0.4	0.03	0.849
A4	4.6	0.511	0.12	0.938	19.69	0.96	25.96	0.986	0.4	0.03	0.881
M1	4.8	0.444	0.14	0.942	20.63	0.943	28.56	0.982	0.38	0.04	0.893
M2	4.29	0.571	0.1	0.926	18.26	0.971	22.85	0.987	0.42	0.03	0.865
C1	3.42	0.517	0.06	0.82	14.62	0.955	19	0.978	0.4	0.02	0.742
C2	2.94	0.544	0.05	0.783	12.55	0.965	16.04	0.985	0.41	0.01	0.716
C3	2.55	0.618	0.04	0.796	10.81	0.976	12.86	0.985	0.43	0.01	0.744

## Data Availability

The authors confirm that the data supporting the findings of this study are available within the article.
